# PXL: a nucleic acid-binding module of the promyelocytic leukemia protein

**DOI:** 10.1093/nar/gkag966

**Published:** 2026-10-13

**Authors:** Daniel Fairchild, Irina V Semenova, Dane Geddes-Buehre, Yunfeng Li, Renata Szczepaniak, Sandra K Weller, Bing Hao, Irina Bezsonova

**Affiliations:** Department of Molecular Biology and Biophysics, School of Medicine, University of Connecticut, 263 Farmington Avenue, Farmington, CT 06030, United States; Department of Molecular Biology and Biophysics, School of Medicine, University of Connecticut, 263 Farmington Avenue, Farmington, CT 06030, United States; Department of Molecular Biology and Biophysics, School of Medicine, University of Connecticut, 263 Farmington Avenue, Farmington, CT 06030, United States; Department of Molecular Biology and Biophysics, School of Medicine, University of Connecticut, 263 Farmington Avenue, Farmington, CT 06030, United States; Department of Molecular Biology and Biophysics, School of Medicine, University of Connecticut, 263 Farmington Avenue, Farmington, CT 06030, United States; Department of Molecular Biology and Biophysics, School of Medicine, University of Connecticut, 263 Farmington Avenue, Farmington, CT 06030, United States; Department of Molecular Biology and Biophysics, School of Medicine, University of Connecticut, 263 Farmington Avenue, Farmington, CT 06030, United States; Department of Molecular Biology and Biophysics, School of Medicine, University of Connecticut, 263 Farmington Avenue, Farmington, CT 06030, United States

## Abstract

The promyelocytic leukemia protein (PML) is a stress-response factor that assembles into PML nuclear bodies, dynamic subnuclear compartments involved in tumor suppression and antiviral defense. The most abundant isoform, PML-1, has been linked to transcriptional regulation, genome stability, and antiviral responses, yet the molecular basis of these functions remains unclear and is generally attributed to proteins associated with PML nuclear bodies. Here, we report that PML-1 itself harbors a previously structurally uncharacterized nucleic acid-binding domain. The domain, termed PXL, is encoded by exon 9 of the PML gene and is unique to isoform 1. Its structure reveals an exonuclease III fold, but lacks the catalytic residues required for activity, defining PXL as a pseudo-exonuclease. Biochemical, structural, mutational, and cellular analyses demonstrate that PXL specifically binds G-rich oligonucleotides implicated in G-quadruplex formation and displays a strong *in vitro* preference for polyUG RNA. Furthermore, deletion of the PXL domain significantly decreases PML colocalization with G-quadruplexes in cells, downregulates genomic maintenance pathways, and modulates the expression of E2F target and interferon-α response genes. These findings provide a molecular basis for previous observations implicating PML in nucleic acid-associated functions and establish a mechanistic framework for future studies of PML-mediated stress responses, nuclear organization, and gene regulation.

## Graphical Abstract

**Graphical Abstract:**
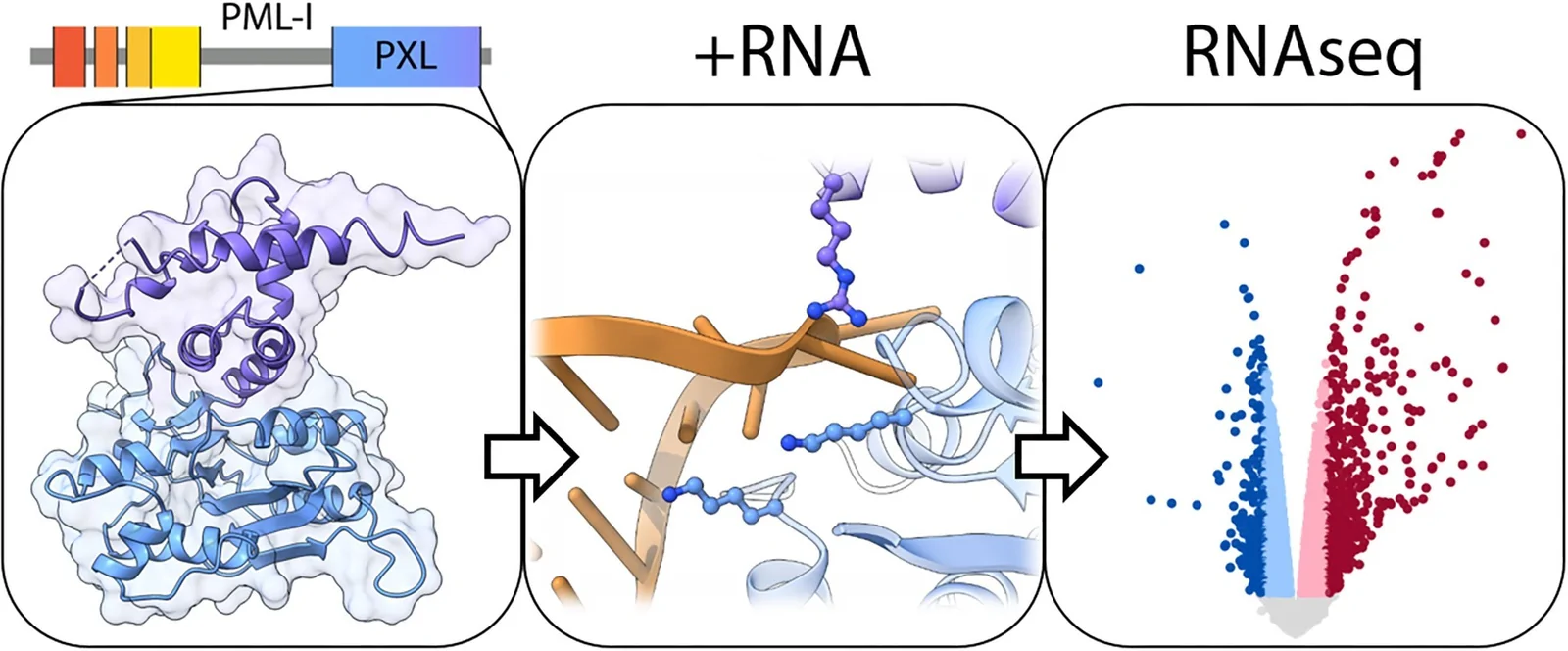

## Introduction

The promyelocytic leukemia protein (PML, or TRIM19) is a SUMO E3 ligase [1] and scaffold protein that organizes spherical subnuclear compartments known as PML nuclear bodies (PML-NBs) [2]. These bodies play key roles in cellular stress responses. PML forms the outer shell of PML-NBs and recruits numerous SUMOylated client proteins, including Sp100, DAXX, transcription factors, and DNA repair proteins, into the interior [3, 4]. These dynamic compartments act as hubs that coordinate transcription, DNA repair, antiviral defense, and genome maintenance [4–6]. Consistent with these roles, PML-NBs have been observed near sites of DNA damage [7], regions of nascent RNA transcription [8], and viral DNA [6]. PML also contributes to the innate antiviral immune response, and many DNA viruses, including herpes simplex viruses, target and disrupt PML-NBs [9–11]. Viral genomes of herpesviruses and adenoviruses are often localized adjacent to PML-NBs, which have been shown to have an inhibitory effect on viral replication [3, 9]. Despite these well-established associations, the molecular mechanisms by which PML-NBs execute these diverse functions remain poorly understood.

There are seven primary PML isoforms (1-7) produced by alternative splicing of the *PML* gene [5]. They share a common N-terminal motif, which consists of RING, B-box1, B-box2, and coiled-coil domains (RBCC), but differ in their C-termini (Fig. 1A). While the RBCC motif is essential for PML-NB formation, the variable C-termini mediate isoform-specific interactions with nuclear components [12]. PML-1 is the most abundant isoform [13]. The isoform-specific functions of PML-1 include regulating transcription factors, targeting nucleoli, maintaining genomic stability, and responding to DNA viruses [14–16]. It is highly expressed in breast cancer, where its levels correlate with poor prognosis and have been reported to contribute to endocrine therapy resistance [13, 17]. ChIP-seq analyses show that, unlike other PML isoforms, PML-1 localizes to promoters of stemness genes, enhancing the stem-like properties of cancer cells [13]. These findings suggest that the PML-1 isoform contains a unique module that directs it to specific genomic loci.

**Figure 1.**
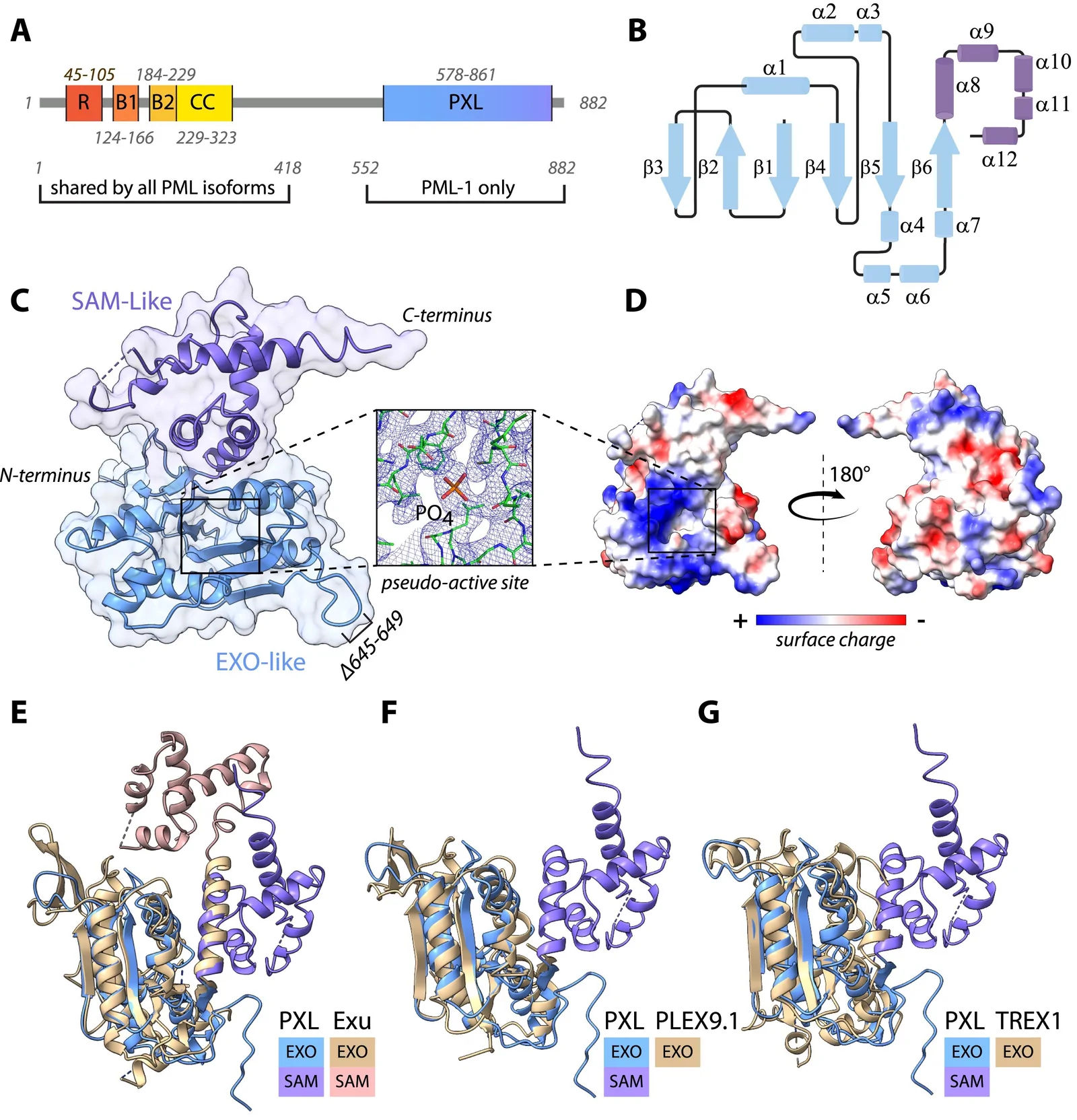
Isoform 1 of PML contains an exonuclease-like domain, PXL. (**A**) Schematic domain organization of PML-1, which consists of N-terminal Ring (**R**), Bbox1 (B1), Bbox2 (B2), and coiled coil domain shared by all PML isoforms, and isoform 1-specific C-terminal PXL domain. (**B**) Secondary structure diagram of PXL. (**C**) Crystal structure of the PXL domain with two subdomains labeled, EXO-like (light blue) and SAM-like (purple). The inset shows the electron density of the pseudo-active site of the EXO sub-domain with the phosphate ion labeled. (**D**) Surface representation of PXL domain in two orientations, colored by surface charge from positive (blue) to negative (red). An extensive positively charged surface patch (blue) surrounds the pseudo-active site (square). (E-G) Structural overlay of PXL and its closest structural homologs found in the Protein Data Bank: (**E**) Exu (Z-score: 19.1, RMSD: 2.7), (**F**) PLEX9.1 (Z-score: 17.9, RMSD: 2.1), and (**G**) TREX1 (Z-score: 15.1, RMSD: 2.7). Structures and sub-domains are color-coded and labeled.

The unique C-terminal sequence of PML-1, encoded by exon 9 of the *PML* gene, includes a putative exonuclease III-like domain [14] (Fig. 1A). An orthologue of PML-1 found in spotted gar has been shown to function as a cytoplasmic exonuclease, where it can suppress retroelements and maintain genomic stability [15]. Interestingly, human PML-1 does not appear to retain its exonuclease function and has adapted a new role. While still able to restrict cytoplasmic retroelements via the ubiquitin-proteasome system, the C-terminal region of PML-1 has been shown to direct the localization of endogenous PML to the nucleolar fibrillar centers in response to genotoxic stress. This results in the formation of large senescence-associated nuclear bodies, which contain nucleolar components [14]. Despite the interesting evolutionary path that PML-1 has undergone and its known roles in senescence and genome stability, it remains unknown how the putative exonuclease-III-like domain of PML-1 drives these functions. To date, nucleic acid binding, or DNase/RNase activity, has not been explicitly demonstrated for human PML-1.

Here, we show that PML-1 contains a pseudo-exonuclease-like domain, PXL (Pseudo eXonuclease-Like), a nucleic-acid–targeting module unique to isoform 1. Structural and functional analyses, including X-ray crystallography, biochemical, biophysical, cellular, and multi-omics assays, reveal that PXL directly and specifically binds single-stranded G-rich RNA and DNA motifs *in vitro* and influences the cellular transcriptome. Together, these findings establish a direct nucleic acid-binding function for PML-1 and provide a mechanistic framework for its roles in transcriptional regulation, stress response, and genome maintenance.

## Materials and methods

### Protein expression and purification

The PXL gene sequence was codon-optimized for bacterial expression and chemically synthesized (Genscript). Ligation-independent cloning was used to insert the PXL gene (578–861∆5) into a pET-based vector containing Tobacco Etch Virus (TEV)-cleavable His6 and SUMO tags. The vector was a gift from Scott Gradia (Addgene plasmid #29 711). To generate PXL(578–861∆5)2K1R/3D, R792D, K678D, and K725D mutations were introduced into the PXL(578–861∆5) sequence using site directed mutagenesis. The PXL(578–861∆5) construct was transformed into the C43 strain of *Escherichia coli* and plated on lysogeny broth (LB) medium with ampicillin. An isolated colony of the transformation was used to inoculate 50 ml of LB broth for an overnight growth at 37°C. From the overnight culture, 25 ml was used to inoculate 1 l of LB medium containing ampicillin, which was grown at 37°C to an OD600 of 0.8 a.u. Protein expression was induced by the addition of 1 mM Isopropyl β–D–1–thiogalactopyranoside(IPTG) and incubated overnight at 18°C. Cell pellets were resuspended in lysis buffer (20 mM NaH_2_PO_4_, pH 8.0, 500 mM NaCl, 5 mM 2-mercaptoethanol (BME)) supplemented with 15 mM imidazole, then lysed by sonication. The insoluble components of the lysate were removed via centrifugation at 15 000 revolutions per minute (rpm) in a Sorvall RC 6 + centrifuge. The soluble lysates were then purified on a HisPur cobalt resin column and eluted using lysis buffer supplemented with 400 mM imidazole. Eluted proteins were dialyzed into buffer containing 20 mM Tris–HCl, pH 7.5, 100 mM NaCl, 5 mM BME, and cleaved with TEV protease overnight at 4°C. After dialysis and cleavage, PXL(578–861∆5) was further purified on a HiTrap Heparin HP affinity column using linear gradient of NaCl (0.1–1.0 M) in 20 mM Tris–HCl, pH 7.5, and 5 mM BME. Eluted fractions were then collected and analyzed by sodium dodecyl sulfate–polyacrylamide gel electrophoresis. Fractions containing purified PXL(578–861∆5) were pooled, and buffer exchanged into 20 mM Tris–HCl, pH 7.5, 100 mM NaCl, and 5 mM BME.

The TREX1 expression plasmid was a gift from Cheryl Arrowsmith (Addgene plasmid # 220 884). TREX1-6xHis was transformed into the Bl21(DE3) strain of *E. coli* and plated on LB agar containing kanamycin. An isolated colony was used to inoculate 50 ml of LB containing kanamycin for an overnight culture at 37°C. From the overnight culture, 25 ml were used to inoculate 1 l of LB containing kanamycin, which was grown at 37°C until it reached an OD_600_ of 0.8 a.u. Protein expression was then induced by the addition of 1 mM IPTG overnight at 18°C. Cell pellets were resuspended in lysis buffer (50 mM NaH_2_PO_4_, 300 mM NaCl, 10 mM imidazole, pH 8.0) and lysed by sonication. The insoluble components of the lysate were removed via centrifugation at 15 000 rpm in a Sorvall RC 6 + centrifuge. The soluble lysates were then purified on a HisPur cobalt resin column and eluted using 300 mM imidazole in lysis buffer (pH 8.0). Eluted proteins were then dialyzed into fast protein liquid chromatography (FPLC) buffer (20 mM Tris–HCl, 150 mM NaCl, pH 8.0) overnight at 4°C, and simultaneously, the 6xHis tag was removed with TEV protease. After dialysis and cleavage, TREX1 was further purified by size-exclusion chromatography using a HiLoad Superdex 75 (Cytiva) column in FPLC buffer.

### X-Ray crystallography

Seed crystals of PXL(578–861∆5) (10 mg/ml) were first obtained in 2 M NaCl, 100 mM Tris–HCl, pH 7.0, and 200 mM MgCl_2_ at 16°C using hanging-drop vapor diffusion and then crushed through shaking with small glass beads using a vortex. Larger PXL(578–861∆5) (10 mg/ml) crystals were then obtained in 2M NaCl, 100 mM Tris–HCl (pH 6.75), and 200 mM MgCl_2_ at 16°C by seeding via hanging-drop vapor diffusion. Crystals were cryoprotected in reservoir solution supplemented with 20% glycerol and flash-cooled in liquid nitrogen. X-ray diffraction data were collected at NSLS-II beamlines 17-ID-1 and 17-ID-2, and processed using Fast DP [18, 19] and autoPROC [20]. The crystals contain one molecule in the asymmetric unit. The structure of PXL(578–861∆5) was determined by molecular replacement in Phaser/CCP4 [21, 22] using the structure model generated by AlphaFold3 [23] as a starting model. The models were refined by alternating cycles of manual rebuilding in Coot and refinement with REFMAC5/CCP4 [22, 24]. Data collection and refinement statistics are summarized in Table 1. Ramachandran statistics were calculated using MolProbity [25], and figures were generated in Chimera [26] and PyMOL (Schrödinger, LLC).

**Table 1.** Summary of crystallographic analysis

Data collection	PXL(578–861∆5)
Wavelength (Å)	0.9793
Space group	*P*3_1_21
Cell dimensions (Å)	
*a, b, c* (Å)	114.34, 114.34, 102.68
*α*, β, γ (°)	90.0, 90.0, 120.0
Resolution (Å)	33.0–3.01 (3.39–3.01)
*R*_sym_ (%)	0.46 (3.75)
Mean (*Ι*/σ*Ι*)	6.0 (1.4)
Completeness (%)	93.3 (67.3)
CC (1/2)	0.992 (0.324)
Multiplicity	12.5 (12.2)
Refinement	
Resolution (Å)	21.61–3.01
No. reflections (|F|>0σ)	9707
*R*_work_/*R*_free_ (%)	16.7/24.1
No. atoms	
Protein	2 069
Water	194
Average B-factors (Å^2^)	
Protein	87.4
Water	57.4
Wilson B-factors (Å^2^)	95.2
Root mean square (R.m.s.) deviations	
Bond lengths (Å)	0.01
Bond angles (°)	1.18
Ramachandran plot (%)	
Favored (%)	100
Allowed (%)	0.0
Outliers (%)	0.0

Values in parentheses are for the highest-resolution shell.

### Exonuclease activity assays

The exonuclease activity binding mixtures contained 500 nM of a linearized 6 kB plasmid with staggered ends, and either 200 nM TREX1 or PXL in 20 mM Tris–HCl, pH 8.0, 100 mM NaCl, 5 mM BME, and 5 mM MgCl_2_ or 10 mM ethylenediaminetetraacetic acid (EDTA). Binding mixtures were incubated for 0, 15, or 30 min, and reactions were stopped by adding loading dye containing sodium dodecyl sulfate (SDS). Binding mixtures were resolved at 100V for 30 min in 0.5% agarose gel in Tris-acetate-EDTA (TAE) buffer and stained with SYBR Safe DNA. Gels were imaged using a Syngene G:Box gel imager.

### Electrophoretic mobility shift assays

The electrophoretic mobility shift assay (EMSA) binding mixtures contained 500 nM 5′ or 3′-AlexaFluor488 labeled nucleotide substrates, 0–3 µM PXL(578–861∆5), 20 mM HEPES, pH 7.3, 150 mM NaCl, 1 mM BME, 2.5 mM MgCl_2_, 100 µg/ml bovine serum albumin, and 6% ficol. Binding mixtures were incubated on ice for 30 min and resolved by gel electrophoresis using a native 5% polyacrylamide Tris-borate-EDTA (TBE) gel. Gels were visualized with the Bio-Rad ChemiDoc MP imaging system.

5′ and 3′-AF-488 labeled oligonucleotides were chemically synthesized (IDT); 5′ AF-488 RNA sequences: 5′-UGUGUGUGUGUGUGUGUGUG-3′, 5′-UGUGUGUGUG-3′, 5′-ACACACACAC-3′, 5′–AAAAAAAAAA-3′, 5′-CCCCCCCCCC-3′, 5′–UUUUUUUUUU-3′; and 5′AF-488 single-stranded DNA (ssDNA) sequences: 5′-TGTGTGTGTG-3′, 5′-ACACACACAC-3′, 5′-AAAAAAAAAA-3′, 5′-GGGGGGGGGG-3′, 5′-CCCCCCCCCC-3′, 5′-TTTTTTTTTT-3′; and 3′ AF-488 double-stranded DNA (dsDNA) sequences: 5′-AGCTATGGCGTCGAA-3′ and 3′- TCGATACCGCAGCTT-5′.

### Fluorescence polarization

FP experiments were performed with 100 nM 5′-AlexaFluor488 labeled nucleotide substrates on a SpectraMax iD5 fluorescence plate reader. Nucleotide substrates were incubated with PXL (578–861∆5) in 20 mM Tris–HCl, pH 7.5, 1 mM BME, 1 mM EDTA, and 150 mM NaCl. The total reaction volume was 15 µl. Duplicates of each titration point were prepared and measured with 485 and 535 nm as the respective excitation and emission wavelengths. The data were fit using nonlinear regression in Prism 10 (GraphPad).

### Cell lines

PML^-/-^ U2OS cells [27] were grown in McCoy’s 5A modified medium supplemented with 10% fetal bovine serum (FBS) and were seeded into a six-well plate at approximately 3 × 10^4^ cells per well. On the following day, cells were washed twice with phosphate buffered saline (PBS), and the media were replaced with Opti-MEM reduced serum media. Cells were transfected with either YFP-WT-PML-1 or YFP-PML-1(1–600) with the addition of pUC119 used as a carrier DNA. Transfections were performed using Lipofectamine 2000 according to the manufacturer’s instructions and carried out over 24 h, after which the transfection medium was replaced with growth medium and the cells were incubated for an additional 24 h. Cells transfected with YFP-WT-PML-1 or YFP-PML-1(1–600) were washed 2× with PBS and exposed to 0.05% Trypsin–EDTA. Trypsinization was halted by adding growth medium supplemented with 10% FBS. Cell suspensions were sorted using the FACSymphony S6 cell sorter (BD Biosciences). Single YFP-positive cells were plated in a 96-well plate and expanded in growth media under selection of 500 µg/ml Geneticin (G418).

### Immunofluorescence

PML^-/-^ U2OS cells [27] expressing either WT YFP-PML-1 or its truncation mutant (residues 1–600) lacking the C-terminal PXL domain, YFP-PML-1-ΔPXL, were seeded into a 24-well plate on coverslips and allowed to recover in growth media for 24 h. After recovery, U2OS cells were washed 3× with PBS and fixed with 2% paraformaldehyde for 20 min at room temperature. Cells were then washed 3× with PBS and permeabilized with 0.5% Triton-x100 in PBS for 10 min. Following permeabilization, the cells were again washed 1× with PBS and incubated in 3% normal goat serum (NGS) at room temperature for 1 h. Cells were then incubated in a 1:1000 dilution of primary antibody [Anti-DNA G-quadruplex (G4) clone 1H6 ZooMAb^®^ mouse monoclonal] in NGS for 1 h at room temperature. After incubation with primary antibody, cells were washed 5× with PBS and incubated with a 1:5000 dilution of secondary antibody (Goat anti-mouse IgG Alexa Fluor™ 594 A-11032) in a dark box for 30 min at room temperature. Cell nuclei were then stained with Hoechst 3342 nuclear stain, followed by 5× PBS washes in a dark box. Coverslips were then mounted on slides using Fluoroshield™ mounting media and stored at 4°C until imaging. Imaging was performed using the BioTek Cytation^TM^ 5 Cell Imaging and Multimode Reader. Sample images were obtained using a 60× Olympus Plan Fluorite objective, 4',6-diamidino-2-phenylindole (DAPI) filter cube (Excitation 377/50 nm Emission 477/60 nm), YFP filter cube (Excitation 500/24 nm Emission 542/27 nm), and Texas Red filter cube (Excitation 586/15 nm Emission 647/57 nm). Cell images were automatically background subtracted and deconvolved using Gen5^TM^ analysis software.

### Cellular analysis

Colocalization analysis of immunofluorescence was performed using the image processing software FIJI [28]. Initially, photos of individual cell nuclei were split into their corresponding red (G-quadruplex) and yellow (PML) channels, and these channels were then subsequently smoothed using FIJI processing commands. To select only YFP-PML NBs in the yellow channel, the image was binarized where any signal intensity threefold greater than or equal to the average YFP nuclear signal is equal to 1, and all other signal is equal to 0. Using the Analyze Particles tool on FIJI, regions of interest (ROI’s) were selected to include areas with a signal value of 1 and total area of 0.1 µm^2^ or greater, where each ROI corresponds to a PML NB. In the red channel, to locate regions of concentrated probe signal intensity, the images were binarized where signal intensity greater than or equal to twofold the diffuse nuclear signal is equal to 1, and all other signal is equal to 0. The binarized red channel intensity was then measured within the ROIs of the yellow channel. Yellow channel ROIs that contained binarized red signal were denoted as colocalized PML NBs.

The principles of determining the colocalization coefficient are similar to those described by Manders and colleagues [29]. Colocalization was defined as the ratio between the number of PML nuclear bodies that contain probe fluorescence (N_c_) and the total number of nuclear bodies (N_t_):


\begin{eqnarray*}
C = \frac{{{{N}_c}}}{{{{N}_t}}}
\end{eqnarray*}

The binarized yellow channel was also utilized for automated counting of the number of PML-NBs in each cell using the Analyze Particles tool on FIJI.

Corrected total cellular fluorescence (CTCF) was calculated from non-binarized images of cell nuclei using the following equation:


\begin{eqnarray*}
\mathrm{CTCF} = ID - \left(A_{\mathrm{nuc}} \times \overline{F}_{\mathrm{bg}}\right)
\end{eqnarray*}

Where *ID* is the integrated density, $A_{\mathrm{nuc}}$ is the total area of the nucleus, and $\overline{F}_{\mathrm{bg}}$ is the average background fluorescence. GraphPad Prism 10 was used to plot individual cellular colocalization coefficients, nuclear body analysis, and CTCF. Unpaired t-tests were used where applicable, and a *P*-value <.05 was considered statistically significant.

### Bulk RNA-seq

PML^-/-^ U2OS, WT U2OS, PML^-/-^ U2OS expressing WT YFP-PML-1 or YFP-PML-1-ΔPXL, were cultured in 100 mm dishes to 90% confluency. Cells were subsequently washed 2× with PBS, resuspended using 0.05% trypsin. Following resuspension, PML^-/-^ U2OS expressing WT YFP-PML-1 or YFP-PML-1-ΔPXL cells were sorted by YFP fluorescence using the FACSymphony S6 cell sorter. Cells with a detected YFP signal were collected separately and used for bulk RNA-sequencing (RNA-seq). CTCF was used to assess YFP-PML-1 and YFP-PML-1ΔPXL protein expression levels (Supplementary Fig. S4C). For all cell lines, 100 000 cells were pelleted at 100 × *g* and washed with PBS. Following washing, cells were pelleted again at 100 × *g* and resuspended in DNA/RNA Shield^TM^ (ZYMO Research) until shipment to PlasmidSaurus (plasmidsaurus.com) for RNA-seq sample preparation and data collection. Total RNA from three biological replicates per experimental group was processed for sequencing using a 3′-end counting RNA-seq workflow. Messenger RNA was converted to complementary DNA by reverse transcription followed by second-strand synthesis. Libraries were generated by tagmentation, indexing, and polymerase chain reaction amplification to enable 3′-end transcript counting. Sequencing was performed on an Illumina NovaSeq platform, generating approximately 10 million ∼90-bp reads per sample. Raw sequencing reads were assessed for quality using FastQC (v0.12.1) and filtered using Fastp (v0.24.0). Filtered reads were aligned to the human reference genome using the STAR aligner (v2.7) [30], with 86%–93% of reads mapping to the reference genome. Gene expression was quantified using the 3′-end counting workflow, and differential gene expression analysis was performed by comparing read counts between experimental groups. Genes with a fold change ≥2 and an adjusted *P*-value ≤.05 were considered significantly differentially expressed. Additional details regarding data processing are available at Plasmidsaurus (https://plasmidsaurus.com).

### Quantitative mass spectrometry-based proteomics

#### Proteomics sample preparation

PML^-/-^ U2OS, WT U2OS, PML^-/-^ U2OS expressing WT YFP-PML-1 or YFP-PML-1-ΔPXL, were cultured in 100 mm dishes to 90% confluency. Cells were subsequently washed 2× with PBS, resuspended using 0.05% trypsin. The resuspended cells samples were then pelleted at 100 × *g* and washed 2× with PBS. The washed cells were then pelleted at 100 × *g* and resuspended in 1× radioimmunoprecipitation assay (RIPA) buffer. Lysed cells were flash frozen and stored at −80°C until analysis.

#### Untargeted protein identification and label-free quantification by liquid chromatography mass spectrometry

Sample lysates were quantified by bicinchoninic acid (BCA) assay (Pierce #23 250) and 100 μg of each sample was aliquoted, adjusted to 2% SDS, and processed with S-Trap columns (ProtiFi, LLC) using a modified commercial protocol. In brief, samples were reduced with dithiothreitol at 25°C for 1 h, alkylated with iodoacetamide for 30 min at 25°C in the dark, acidified with phosphoric acid, and diluted by 6× with binding/wash buffer (90% methanol, 100 mM triethylammonium bicarbonate (TEAB)). Samples were loaded onto S-trap micro columns, washed with a total of 600 μl binding/wash buffer, and digested at 25°C overnight with trypsin/LysC at 1:10 enzyme:protein (w/w, Promega #V5073). Peptides were eluted with sequential washes of 50 mM ammonium bicarbonate, 0.2% formic acid in water, and 0.2% formic acid in 50% acetonitrile, dried to completion, desalted by reverse-phase chromatography using Pierce peptide desalting spin columns (Thermo Fisher part #89 852), dried to completion again, and resuspended in Solvent A (0.1% formic acid in Fisher Optima LC/MS grade water). Final peptide concentration was quantified by A_280_ absorbance and samples were normalized by dilution to a common concentration for injection.

Peptides were analyzed using a Thermo Scientific Ultimate 3000 RSLCnano ultra-high performance liquid chromatography (UPLC) system coupled to a Bruker timsTOF HT mass spectrometer. Each sample was injected onto a nanoEase M/Z Peptide BEH C18 column (1.7 μm, 75 μm x 250 mm, Waters Corporation) and separated by reversed-phase UPLC using a gradient of 4%–30% Solvent B (0.1% formic acid in Fisher Optima LC/MS grade acetonitrile) over a 50-min gradient at 300 nL/min flow, followed by a ramp of 30%–90% Solvent B over 10 min, for a 60-min total gradient. Peptides were eluted directly into the HT using positive mode nanoflow electrospray ionization with a capillary voltage of 1500 V. Data were acquired using a modified DIA-PaSEF method with the following parameters: MS scans were taken over an m/z range of 100–1700, and a 1/K0 range of 0.7–1.4 V s/cm^2^, with a rolling average of 10 scans. TIMS was enabled, with an intensity threshold of 5000, a ramp time of 100 ms, and an accumulation time of 2 ms. MS2 scans were taken over a mass range of 400–1201 Da in 25 Da windows with a 1 Da overlap, and over a mobility range of 0.6–1.43 1/K0. Cycle time was 1.8 s. Fragmentation energy was ramped from 20–59 eV over the mobility space.

Peptides and proteins were identified using Spectronaut (v19.9.250324, Biognosys) in directDIA mode, searching against the *Homo sapiens* reference proteome (UP000005640, downloaded from UniProt on 3 June 2024), and the MaxQuant common contaminants database. Protease specificity was set to trypsin/P with a maximum of 2 missed cleavages, considering peptides from 7–52 residues in length. Up to five variable modifications per peptide were considered from the following list: protein N-term acetylation, Asn/Gln deamidation, Gln conversion to pyro-Glu, and Met oxidation. Cys carbamidomethylation was set as a fixed modification. All false discovery rate (FDR) thresholds were 1% based on the target-decoy method. Quantification was defined as area under the curve at the MS1 level, with no cross-run normalization applied. All other parameters were left at default values. Subsequent data visualization and analysis was done with msDiaLogue [31] (https://github.com/uconn-scs/msDiaLogue) and GraphPad Prism 10.

## Results

### Isoform 1 of PML contains an exonuclease-like domain

Protein sequence analyses of PML isoforms [32] in combination with AlphaFold [23, 33] predict a structured domain within the C-terminal region of PML-1 (Fig. 1A and Supplementary Fig. S1). This region has previously been predicted to contain an exonuclease III-like domain [14, 15].

To further characterize the putative nuclease domain, we solved its structure by X-ray crystallography (Fig. 1B–D). The crystallized PML-1 fragment included residues 578–861 with a short deletion (645-FFSIY-649), which significantly improved the domain’s solubility. The structure was refined to 3 Å resolution with R_free_ of 24.1% and an R factor of 16.7% (Table 1), revealing two subdomains: an α/β fold resembling 3′–5′ DEDD family exonucleases [34] (EXO-like), and a helical bundle similar to Sterile Alpha Motif (SAM-like) (SCOP 47768). The domain contains a central six-stranded β-sheet (β3–β2–β1–β4–β5–β6), with both parallel and antiparallel strands sandwiched between helical elements α1, α2, α3, and α4, α5, α7, α8 on each side, forming a typical EXO-like domain (Fig. 1B and C). Immediately following the β6 strand, a 22 Å-long helix, α8, extends into the SAM-like region, where it contributes to a five-helix bundle with α9–α12 helices (Fig. 1B and C). Electrostatic surface analysis revealed an extensive positively charged patch located on one face of the domain (Fig. 1D), consistent with the nucleic acid-binding properties of nuclease-like folds.

Structural homologues of the domain were identified using DALI [35], and revealed significant similarity to 3′–5′ DEDD family nucleases, including exonuclease domains of several DNA polymerases. The top hits were *Drosophila* pseudo-exonuclease Exuperantia (Exu) [36] (PDB:5L80, 11% sequence homology), zebra fish 3′–5′ exonuclease Plex9.1 [15] (PDB:9MRC, 15% sequence homology) and human 3′–5′ exonuclease TREX1 [37] (PDB:7TQP, 10% sequence homology) (Fig. 1E–G). Among structural homologues, only the pseudo-exonuclease Exu has a similar EXO/SAM arrangement. Due to high structural similarity to a pseudo-exonuclease, we designate this PML-1 domain Pseudo-eXonuclease-Like domain, or PXL (pixel).

Interestingly, the relative orientation of the EXO and SAM domains differs between the reported Exu structure and the PXL, suggesting that the extender helix and the loop connecting the EXO and SAM domains are sufficiently flexible to permit domain reorientation. In PXL, this orientation is stabilized by contacts between the connector helix α8 and the α9 helix of the SAM domain, a feature absent in Exu, which instead contains a much shorter connector helix (Supplementary Fig. S2). In addition, unlike PXL, Exu forms a dimer through an interface involving the SAM domain and a β-hairpin insertion within the EXO domain. This β-hairpin is conserved in Exu and PLEX9.1 but is absent from PXL.

### PXL lacks nuclease activity

Structural similarity between PXL and exonucleases raises the possibility that PXL may function as an exonuclease. Canonical 3′–5′ exonucleases catalyze processive hydrolysis of nucleic acids from the 3′ end *via* a two-metal ion-dependent mechanism [34]. DEDD exonucleases share a conserved catalytic core with a β1–β2–β3–α1–β4–α2–β5–α3 fold and an active site formed by four conserved acidic residues (DEDD), often followed by a basic residue (H or Y), which coordinate magnesium ions and mediate hydrolysis [34].

Despite this structural similarity, sequence analysis argues against a catalytic role for PXL. Multiple sequence alignment of PXL with a pseudo-exonuclease Exu and catalytically active exonucleases TREX1 and Plex9.1 reveals that the canonical DEDD residues are not conserved in PXL and Exu but are preserved in active exonucleases (Fig. 2A). Only one of the four DEDD residues, D614, is retained in PXL (Fig. 2A and B). The absence of conserved metal-coordinating residues indicates that the PXL domain of PML-1 is unlikely to function as an exonuclease.

**Figure 2.**
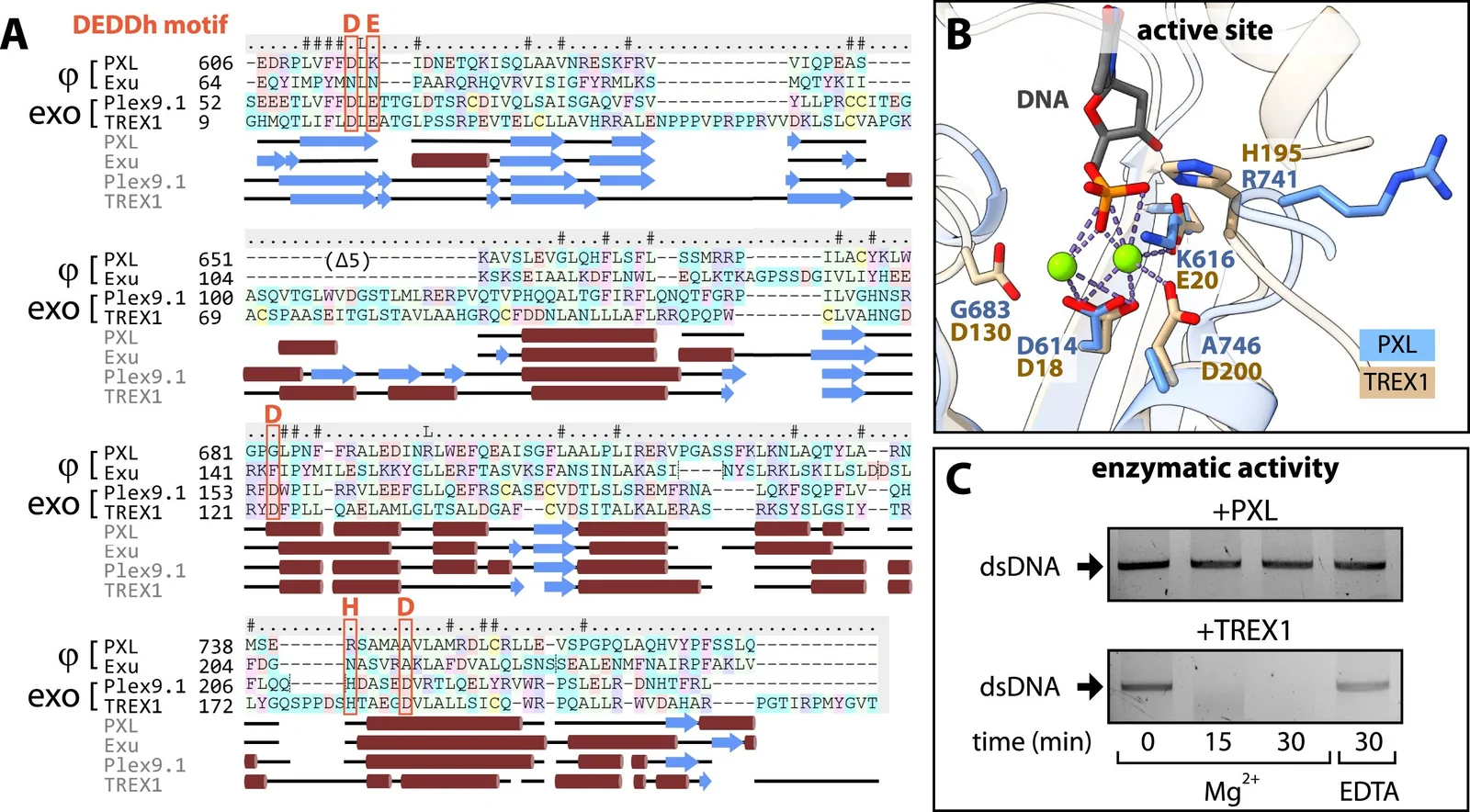
PXL is not an active exonuclease. (**A**) Multiple sequence alignment of the human PML-1 PXL domain, the *drosophila* pseudo-nuclease Exu, zebrafish PML-like exon 9 (Plex9.1), and human TREX1. Predicted secondary structural elements are shown underneath the multiple sequence alignment, alpha helices are depicted as red cylinders, and beta strands as blue arrows. The conserved DEDD nuclease active site is outlined in red. (**B**) Overlay of TREX1 (tan) and PXL (blue) active/pseudo active site. TREX1 catalytic residues and PXL aligned residues are represented as sticks. TREX1 catalytic residues coordinate magnesium ions (green) to facilitate nucleic acid (gray) cleavage. (**C**) Nuclease activity assay showing TREX1 cleaving dsDNA in a metal-dependent manner over time. PXL does not show exonuclease activity.

To experimentally confirm that PXL lacks exonuclease activity, we conducted an *in vitro* activity assay using TREX1 as a positive control (Fig. 2C). As expected, TREX1 completely degraded the dsDNA substrate in ∼30 min, and its activity depended on the presence of magnesium. Under the same experimental conditions, PXL demonstrated no detectable nuclease activity, thus showing that, unlike TREX1, PXL does not facilitate nucleic acid cleavage through coordination of magnesium ions.

Consistent with the sequence analysis, the crystal structure of PXL shows no evidence of magnesium ions in the active site despite the presence of magnesium salt in the crystallization solution. Instead, residual electron density is observed buried deep in the pseudo-active site between β1 and a loop connecting the β4 and α2 secondary structure elements. This density is too large to accommodate two Mg^2+^ ions but is well fit by a single phosphate group and is likely an artifact of the purification conditions (Fig. 1C inset).

### PXL selectively binds G-rich ssRNA and ssDNA sequences

Building on the observation that PXL, like its homolog Exu, is a pseudo-exonuclease, we next tested whether the PXL domain can directly interact with nucleic acids using EMSAs. In the assays, 5′-AF488-conjugated DNA and RNA oligonucleotides were incubated with increasing concentrations of PXL in the presence of 5 mM Mg^2+^. EMSAs revealed that PXL has a clear preference for RNA over ssDNA of the same oligonucleotide composition (Fig. 3A and B) and does not bind dsDNA (Supplementary Fig. S3). Furthermore, PXL has strong RNA sequence specificity. It efficiently binds poly(UG) RNA previously reported as a preferred RNA substrate of Exu [36], but not poly(A), poly(C), or poly(AC), and only weakly binds poly(U) RNA. Interestingly, PXL’s poly(UG) affinity further increased with the increasing number of (UG) repeats, suggesting that there is a minimal size requirement for optimal PXL binding (Fig. 3B). Technical limitations prevented the use of poly(G) RNA, but poly(G) ssDNA effectively associated with PXL in EMSA assays (Fig. 3A). Despite the presence of Mg^2+^ during incubation, no exonuclease activity was detected for any of the tested substrates. These data suggest that PXL can bind RNA and ssDNA *in vitro* and prefers G-rich motifs.

**Figure 3.**
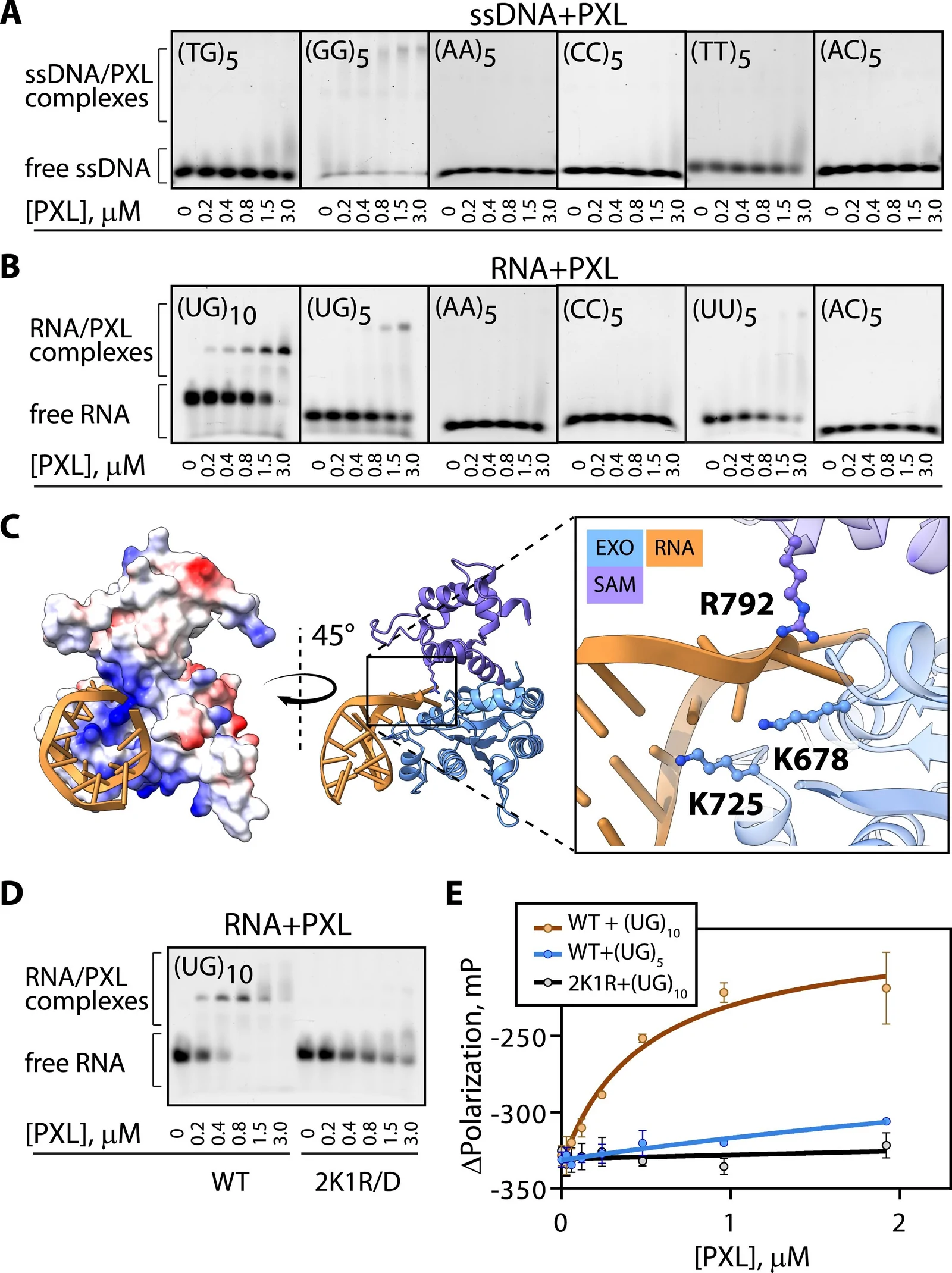
PXL binds to specific G-rich nucleic acid sequences using positively charged surface exposed residues. (**A, B**) EMSA of PXL and different (**A**) ssDNA/(**B**) RNA substrates. PXL demonstrates a clear preference for pUG RNA sequences and G-rich ssDNA motifs. (**C**) AlphaFold3 predicted model of PXL binding UG_(10)_ (yellow). Positively charged residues located on both the SAM-like and EXO-like subdomains used to generate PXL 2K1R/3D mutant are represented as ball and stick in the inset. (**D**) EMSA assay comparing binding of PXL and PXL 2K1R/3D to UG_(10)_ RNA. Mutation of positively charged residues on surface of PXL significantly reduces binding efficiency. (**E**) Fluorescence polarization binding curves of PXL and PXL (2K1R/3D) on poly(UG) sequences of different lengths.

The EMSA results were further validated with the fluorescence polarization binding assays (Supplementary Fig. S3). In these assays, PXL demonstrated robust nanomolar binding affinity for 20-nucleotide-long RNA (UG)_10_ (477 ± 142 nM) and weaker affinity for (UG)_5_ RNA and (GG)_5_ ssDNA oligonucleotides. No binding to dsDNA was detected.

Poly(UG) RNA motifs have been reported to form stable four-stranded helical quadruplex structures, called pUG-folds, stabilized by hydrogen bonding between tetrads of guanine bases [38–41]. Similar structures are formed by poly(G) repeats of ssDNA (G-quadruplexes) [42]. The observed preference of PXL for G-rich motifs capable of forming these structures strongly suggests that PXL recognizes not only specific oligonucleotide sequences but also has further specificity for their secondary structure. This is further supported by previous reports of PML-NBs found adjacent to or overlapping with G-quadruplex structures in cells [43]. Importantly, a minimal (UG)_12_ motif is required for the formation of a stable pUG-fold [41]. Since a slightly shorter (UG)_10_ substrate was used here, the ∼500 nM binding affinity measured in the FP experiments might be even stronger for longer poly(UG) RNA and more stable pUG-folds, which is supported by the dramatically increased affinity for (UG)_10_ compared to (UG)_5_ substrates (Fig. 3E).

### PXL’s positively charged surface is required for its nucleic acid-binding

The PXL domain contains a positively charged surface surrounding its pseudo-active site (Fig. 1D), making it a likely site for nucleotide binding. This hypothesis is supported by an AlphaFold model of PXL in complex with (UG)_10_ RNA (Fig. 3C). To test whether this surface mediates RNA binding, we mutated positively charged residues that constitute the putative RNA-binding site (Fig. 3C inset) and examined the RNA-binding ability of the resulting mutant.

Residues K678, K725, and R792, located on loops of the EXO-like domain between β4–α2 and α4–α5, as well as on α9 of the SAM-like domain, were selected based on the complex model and mutated to aspartates (PXL 2K1R/3D). An EMSA assay comparing WT PXL and the 2K1R/3D mutant showed that these mutations abolished RNA binding (Fig. 3D). These results were further validated using FP assays, which confirmed that substrate binding was effectively eliminated by 2K1R/3D mutations (Fig. 3E).

Together, these data demonstrate that the positively charged surface surrounding the pseudo-active site of PXL is required for its nucleic acid-binding activity.

### PXL enhances the colocalization of PML-NBs with G-quadruplex-rich regions in the nucleus

Previously, PML-NBs have been reported to localize adjacent to G-quadruplex structures in cells [43].To probe whether the affinity of the PXL domain for G-rich substrates contributes to this association, we examined the effect of PXL deletion on the colocalization of PML-NBs with G-quadruplex-rich regions in U2OS cells (Fig. 4).

**Figure 4.**
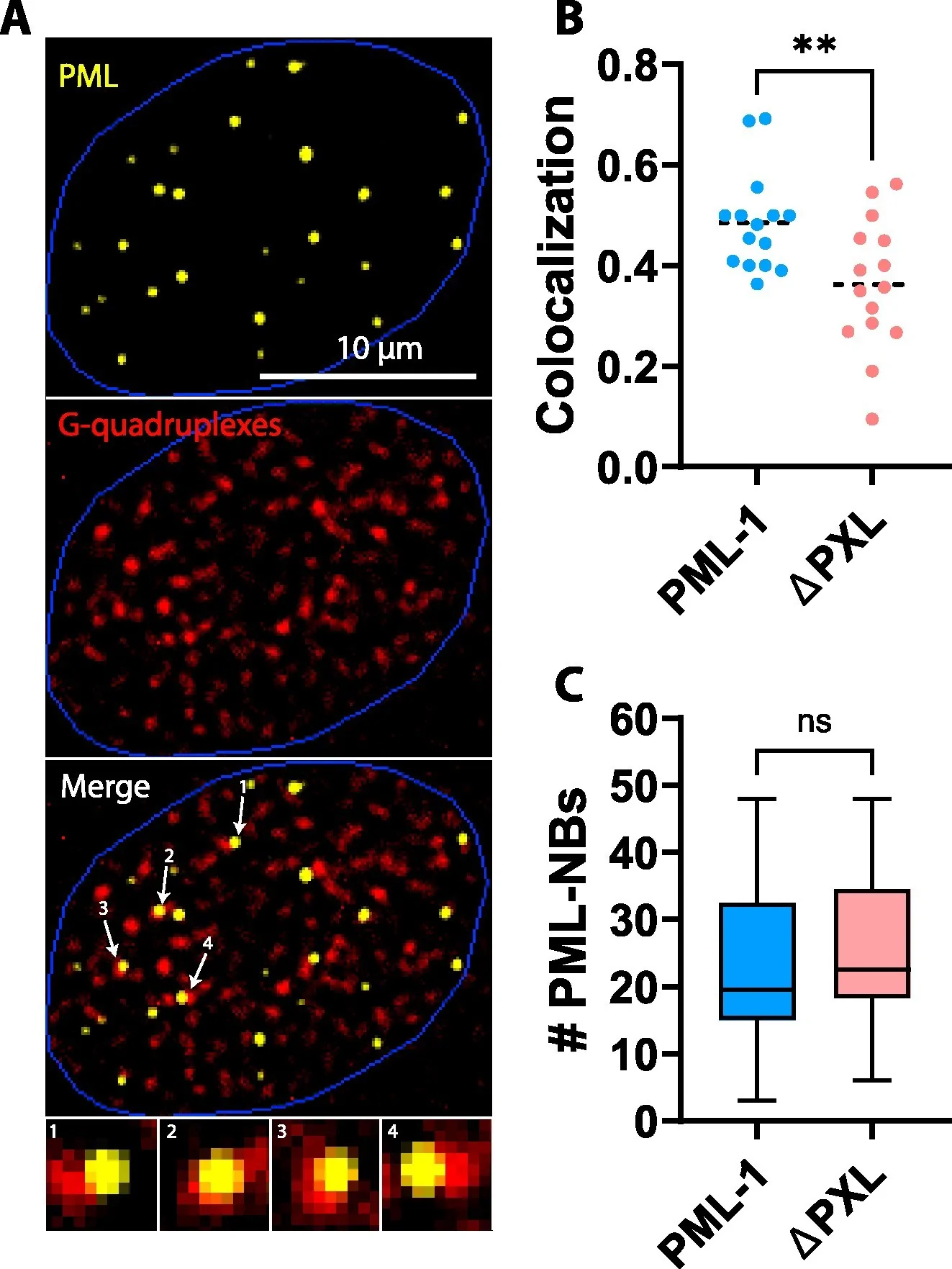
PXL impacts the colocalization of PML-NBs and G-quadruplex structures in cells. (**A**) Representative image panels showing PML NBs (yellow) overlapping with G-quadruplexes (red) in the nucleus of a U2OS cell. Specific examples of overlap are indicated by numbered white arrows in the merge panel. (**B**) Quantification of PML-NB/G-quadruplex overlap for both PML-1 and PML-1∆PXL. (**C**) PML nuclear body counts per cell for both PML-1 and PML-1∆PXL.

For this, PML knockout U2OS cell lines [27] were stably transfected with either YFP-labeled full-length PML-1 or its truncated mutant lacking the PXL domain. Both WT- and ∆PXL-expressing cells formed PML-NBs, indicating that PML-1 is sufficient for nuclear body formation and that the PXL domain is not necessary for this process (Supplementary Fig. S4A and B). G-quadruplex structures were detected using the BG4 (MABE917, *MilliporeSigma*) anti-G-quadruplex antibody, followed by an AF594-conjugated secondary antibody (A-11032, *ThermoFisher Scientific*).

Colocalization between PML-NBs and G-quadruplex-rich regions was defined as an overlap in signal between the YFP and AF594 fluorescence signals (Fig. 4A). Consistent with previous reports, WT PML-1 nuclear bodies showed substantial colocalization with G-quadruplexes (C = 0.49 ± 0.10). In contrast, deletion of the PXL domain significantly reduced colocalization (C = 0.36 ± 0.13), while the number of PML-NBs remained the same (Fig. 4B and C). This indicates that the PXL domain contributes to the efficient association of PML-NBs with G-quadruplex-rich regions in cells. Overall, the differences observed in colocalization between G-quadruplexes and PML-NBs with and without the PXL domain are in good agreement with the preferential binding of the PXL domain to G-quadruplex-forming oligonucleotides observed *in vitro* (Fig. 3).

### PXL influences the transcriptome

Given the sequence-specific nucleic acid-binding properties of the PXL domain, together with the established roles of pUG RNA motifs and G-quadruplexes in gene regulation [38, 42], we hypothesized that PXL contributes to transcriptional regulation. To investigate the impact of the PXL domain on the cellular transcriptome, we performed an RNA-seq study complemented by quantitative proteomics to assess changes in both RNA and protein abundance.

Transcriptomes from four U2OS cell lines were analyzed and compared: WT U2OS cells expressing endogenous PML isoforms (WT), PML-KO cells lacking PML [27], PML-KO cells reconstituted with isoform 1 (PML-1), and PML-KO cells expressing PML-1 lacking residues 601–882 (∆PXL). These comparisons revealed significant changes in RNA abundance across the cell lines, affecting both protein-coding genes and long non-coding RNAs (lncRNAs), consistent with the proposed role of PML-NBs as both transcriptional co-activators and repressors [44] (Fig. 5A and Supplementary Fig. S5).

**Figure 5.**
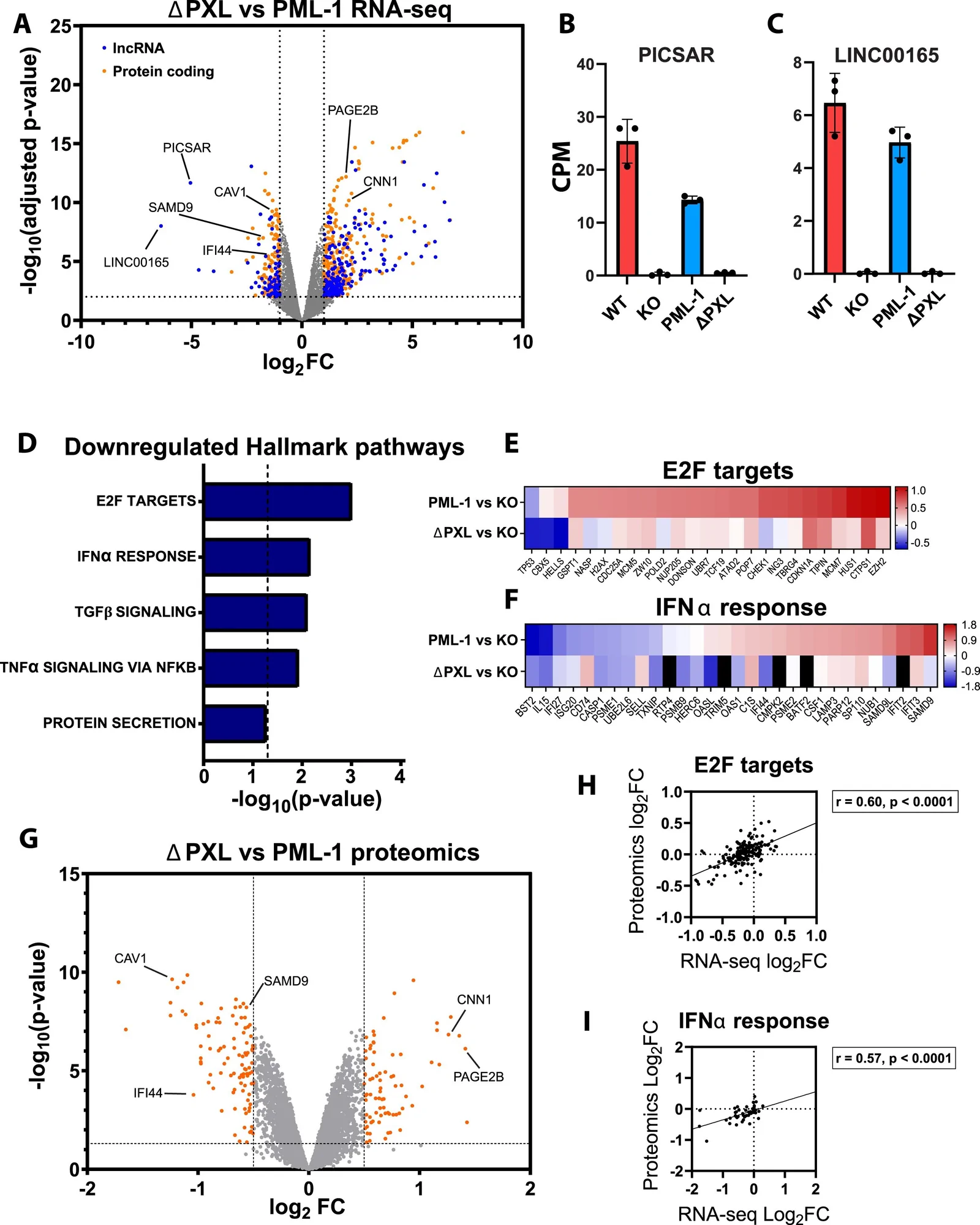
PXL affects the transcriptomic and proteomic profiles of important cellular pathways. (**A**) Volcano plot of gene expression profiles comparing U2OS cells expressing PML-1∆PXL to PML-1. (**B, C**) Abundance counts of lncRNAs PICSAR and LINC00165 in WT U2OS, PML^-/-^, PML-1, and PML-1∆PXL cells. (**D**) Negatively regulated hallmark pathway gene sets identified through gene set enrichment analysis (GSEA), the dashed line is the significance threshold (*P* < 0.05). (**E, F**) Heatmap showing changes in RNA abundance due to PXL deletion of E2F target and interferon-α response gene relative to PML^-/-^ cells. (**G**) Volcano plot of protein abundance changes from mass-spectrometry profiles comparing U2OS cells expressing PML-1∆PXL to PML-1. (**H, I**) Protein–RNA concordance plots showing the correlation between RNA and protein abundance across top negatively regulated hallmark gene sets.

To specifically evaluate the contribution of the PXL domain, we compared the transcriptomes of PML-1 and ∆PXL-expressing cells. Notably, lncRNAs were among the top downregulated transcripts in ∆PXL cells relative to the PML-1 cells. For instance, the oncogenic lncRNAs PICSAR and LINC00165 [44, 45] showed pronounced PXL-dependent expression (Fig. 5B and C). Both transcripts were highly expressed in WT U2OS cells and in cells expressing PML-1, but were substantially reduced in both PML KO and ∆PXL cells. These findings indicate that full-length PML-1, but not the ∆PXL mutant, restores PICSAR and LNC00165 expression following PML depletion. However, these findings may require additional validation using orthogonal approaches. Nonetheless, extensive PXL-dependent changes in lncRNA levels suggest that the PXL domain may contribute to the regulation of lncRNA expression.

To identify biological processes regulated by the PXL domain, we performed Gene Ontology enrichment analysis of PXL-dependent protein-coding genes (Supplementary Fig. S5A). Loss of the PXL domain resulted in downregulation of genes involved in telomere maintenance, DNA repair, and DNA replication pathways, while genes associated with responses to stimuli and environment were upregulated (Supplementary Fig. S5B and C). These findings are consistent with established functions of PML-NBs in genome maintenance and cellular stress responses [7, 14, 15, 46].

To identify coordinated biological processes regulated by PXL, we next performed GSEA using the MSigDB Hallmark gene sets [47, 48]. Deletion of the PXL domain significantly downregulated multiple pathways, including E2F targets and the interferon-α response (*P* < 0.05; Fig. 5D and Supplementary Fig. S6). Consistent with this observation, deletion of the PXL domain reduced E2F target and interferon-α response gene expression, whereas expression of full-length PML-1 increased E2F target and interferon-α response gene expression relative to PML-KO cells (Fig. 5E and F). These data suggest that the PXL domain of PML-1 can positively regulate the interferon-α response and E2F target gene expression, whereas PML isoforms lacking the PXL domain may exert the opposite effect. This is in good agreement with PML-4, an isoform lacking the PXL domain that has previously been shown to repress E2F target genes in human fibroblasts [49].

To determine whether these transcriptomic changes were reflected at the protein level, we performed quantitative mass spectrometry-based proteomics using whole-cell lysates (Fig. 5G). We compared changes in RNA and protein abundance for genes within the E2F targets and the interferon-α response Hallmark gene sets identified by transcriptomic GSEA. Protein–RNA concordance plots revealed strong positive correlations between transcript and protein abundance changes, with statistically significant Pearson correlation coefficients for each pathway (Fig. 5H and I), providing additional validation of RNA-seq results. These multi-omics analyses demonstrate that PML-1 impacts gene expression in a PXL-dependent manner at both transcript and protein levels.

Overall, these observations are consistent with the reported function of the closest structural homolog of PXL, Exu, which binds specific RNA secondary structures, including UG motifs, and regulates RNA expression in *Drosophila*. The shared structural and functional properties of these evolutionarily distant proteins raise the intriguing possibility that the catalytically inactive EXO-SAM fold may represent an ancient nucleic acid-binding module that has been repurposed to regulate gene expression in diverse metazoan lineages.

## Discussion

PML-1 is the most abundantly expressed PML isoform in humans and is known to play roles in the anti-viral response, nucleolar targeting, cell senescence, and the maintenance of genomic stability; however, the molecular mechanisms underlying these functions remain poorly understood. Here, we present the first crystal structure of the isoform-defining C-terminal domain of PML-1 and call it PXL (pixel). This region was previously predicted to contain a putative exonuclease III-like domain [14]. We show that it has a DEDDh exonuclease structural fold but lacks the conserved active site and nuclease activity. Instead, we demonstrate that PXL can directly interact with nucleic acids and regulate the cellular transcriptome. Although PML has been previously implicated in transcriptional regulation through the resident proteins of PML-nuclear bodies, some of which are transcription factors [4], this work, for the first time, provides strong evidence that PML-1 itself can directly and specifically interact with nucleic acids.

Interestingly, PXL’s structural homologues Exu and TREX1 homo-dimerize through their EXO domains [36, 37], meanwhile the crystal structure of PLEX9.1 (PDB: 9MRC) appears as a monomer. Exu requires homodimerization for its *in vivo* function; however, its affinity for RNA substrates does not significantly change upon the introduction of dimer-breaking mutations. Currently, we have no evidence to suggest that the PXL domain forms a dimer. On the contrary, the crystal structure of PXL is monomeric, and its comparison with Exu reveals that structural elements that mediate Exu dimerization, specifically, the large β-hairpin insert into the loop connecting β3 and α1 elements of the EXO fold (Fig. 1C), are absent from PXL, where it is replaced by a much shorter loop. These structural differences argue against a conserved dimerization mechanism. However, because five residues within this loop were removed to improve the solubility of the isolated PXL domain, dimerization cannot be completely excluded. Furthermore, full-length PML, like other TRIM family proteins, is expected to dimerize through its coiled-coil region [50], raising the possibility that two PXL domains are brought into close proximity in the context of the full-length protein. Whether PXL dimerization occurs *in vivo* and whether it contributes to nucleic acid recognition or PML function remain important questions for future investigation.

We show that PXL binds nucleic acids in a sequence-specific manner, with the highest affinity for ssRNA containing UG repeats and weaker binding to ssDNA G-repeats, consistent with recognition of structured RNA. This poly(UG) specificity mirrors that of PXL’s closest structural homologue, *Drosophila* Exu, a regulator of mRNA localization and expression in early embryos [36]. The shared preference for UG-rich RNA likely arises, at least in part, from the conserved SAM-like subdomain, a feature present in both PXL and Exu but absent from canonical DEDDh exonucleases. These findings raise the possibility that the catalytically inactive EXO-SAM fold has been evolutionarily repurposed as a conserved nucleic acid-binding module across diverse metazoan lineages. Further biochemical, structural, and evolutionary analyses of EXO-SAM-containing proteins from diverse organisms will be required to validate this model and determine the extent to which this nucleic acid-binding function has been conserved.

Poly(UG) repeats are often enriched near 5′- and 3′-splice sites of pre-mRNA and are known in C.*elegans* to form a unique G4-quadruplex structure called the pUG fold, which marks the 3′ end of mRNA as vectors for gene silencing [40]. Additionally, G-repeat tetrads in DNA are known to form G-quadruplex folds [42]; these quadruplexes can be intrastrand or interstrand and are biologically significant in processes such as replication, transcription, and telomere stability. Notably, G4-quadruplex DNA and RNA have been shown to colocalize with PML-NBs [43]. Here, by comparing colocalization of PML-NBs with G-quadruplexes in the presence and absence of the PXL domain, we demonstrate that PXL facilitates this colocalization. This observation further supports that PXL may engage quadruplex-forming nucleic acids as part of its gene regulatory function.

Although we show that PXL, like Exu, preferentially associates with poly(UG) motifs and exhibits higher affinity for longer RNA substrates, several mechanisms and functional consequences of these interactions are possible. The higher affinity for longer substrates may simply reflect an additive effect, where longer RNAs provide multiple short linear binding sites for PXL. Alternatively, PXL may recognize higher-order RNA structure rather than a linear sequence motif. In this scenario, PXL could either stabilize RNA secondary structure or promote its remodeling. Structural characterization of Exu and PXL in complexes with RNA might be necessary to start tackling these important mechanistic questions.

In line with specificity for structured regulatory repeats and the previously described role of PML as a transcription factor, our transcriptomic data demonstrate that the PXL domain of PML-1 participates in transcriptional regulation, as there is a marked difference in transcriptomic profiles between cells expressing WT PML-1 and those expressing PML-1∆PXL. Remarkably, increased expression of isoform 1 of PML, specifically, was recently linked to a poor prognosis in estrogen receptor-positive breast cancers, and loss of PML was shown to reduce breast cancer stem cell-related gene expression, suggesting that PML-1 can be a promising anti-cancer therapeutic target [13].

In agreement with these reports, our transcriptomic data show that deletion of the PXL domain from PML-1 can impact the abundance of RNA transcripts, many of which contribute to cell fate-decisions such as E2F transcription factor targets. PML-NBs are thought to recruit E2F transcription factors into high concentrations of histone deacetylases and other PML-NB-associated proteins, leading to p53-induced senescence [49]. Since E2F transcription factors and PML-NBs are known to associate, it is conceivable that the PXL domain of PML-1 may act as a regulatory element for E2F target gene transcription.

Additionally, our data indicate that the PXL domain influences the interferon-α response. Interferon-α is a potent inducer of PML-NBs, promoting their formation and the sequestration of viral genomes in primary sympathetic neurons [51]. Consistent with this connection, both our transcriptomic and proteomic analyses revealed a large decrease in expression of the interferon-induced gene 44 (IFI44) in the absence of the PXL domain (Fig. 5A and G). Interestingly, IFI44 expression is induced by the G4-quadruplex stabilizing ligand pyridostatin [52]. Together with our observations that PXL preferentially binds G-rich nucleic acids and promotes PML-NB association with G-quadruplex-rich regions, these findings raise the possibility that PXL promotes IFI44 expression through recognition of G-quadruplex structures.

Our data revealed several disease-relevant genes that are differentially expressed at the RNA and protein levels in a PXL-dependent manner (Fig. 5). For example, caveolae assembly protein (CAV1) and the growth repressor gene sterile alpha motif domain-containing protein 9 (SAMD9) are downregulated in PXL-deficient U2OS cell lines. The dysregulation of CAV1 and SAMD9 has been linked to diseases such as non-alcoholic fatty liver disease and MIRAGE syndrome, respectively [53, 54]. Interestingly, overexpression of both CAV1 and PML are correlated with poor prognosis and survival rate in pancreatic adenocarcinoma patients [55]. The loss of the PXL domain also leads to the increased expression of calponin-1 (CNN1) and tumor antigen PAGE2B. CNN1 expression is typically decreased in bladder cancer, and overexpression can repress the progression of bladder cancer cells [56], while PAGE2B is overexpressed in tumor cells and is a potential therapeutic target for the treatment of endometrial cancer [57].

While the mechanisms by which PML regulates gene expression are likely complex, our findings indicate that the PXL domain contributes substantially to PML’s function as a transcriptional regulator.

Together, this work identifies the PXL domain as a structurally distinct nucleic acid-binding module of PML-1 that links selective recognition of structured nucleic acids to transcriptional regulation, with broad implications for understanding PML function in genome regulation, antiviral defense, and cancer biology.

## Supplementary Material

gkag966_Supplemental_Files

## Data Availability

The bacterial and mammalian expression plasmids utilized in this work have been deposited in and are available from the Addgene database: PML-1(578-861) (#253216), pLNGY-PML.I (#256798), pLNGY-PML.I(1-600) (#256799). An additional PXL(2K1R/3D) mammalian expression construct has also been deposited: pLNGY-PML.I-2K1R-3D (#260897). All other data is available upon request. The atomic coordinates and structure factors of PXL(578-861∆5) have been deposited in the Protein Data Bank (https://www.rcsb.org/) with PDB DOI: https://doi.org/10.2210/pdb10TH/pdb. The RNA sequencing data have been deposited in the Sequence Read Archive (SRA) under accession number PRJNA1423671. The mass-spectrometry proteomics dataset has been deposited into the PRIDE database (PXD082297).
