# Supplementary material for: PXL: a nucleic acid-binding module of the promyelocytic leukemia protein
Source: Nucleic Acids Res. 2026 Oct 13;54(19):gkag966. doi: 10.1093/nar/gkag966 (PMC13659232; doi:10.1093/nar/gkag966)
Supplement: Supplementary file 1 [file gkag966_supplemental_files.zip › Supplementary-Data.docx]

**PXL: A Nucleic Acid–Binding Module of Promyelocytic Leukemia Protein**

Daniel Fairchild, Irina V. Semenova, Dane Geddes-Buehre, Yunfeng Li, Renata Szczepaniak, Sandra K. Weller, Bing Hao, Irina Bezsonova*

Department of Molecular Biology and Biophysics, School of Medicine, University of Connecticut, 263 Farmington Avenue, Farmington, CT, 06030, United States

*Correspondence to [bezsonova@uchc.edu](mailto:bezsonova@uchc.edu)

Supplementary Information

**Supplementary figure S1: The unique C-terminus of PML-1 is predicted to contain an ordered domain.**

**(A)** IUpred3 disorder predictions of all full length PML isoforms. Isoform specific C-termini begin at residue number 419 (vertical red line). PML-1 is predicted to contain an ordered region in its unique C-terminus. **(B)** AlphaFold3 predictions of PML-1 C-terminus (top) and PML-(2-7) (bottom).

**Supplementary figure S2: PXL and Exu SAM-like domains share secondary structure but differ in orientation relative to the Exo-like domain.**

**(A)** Overlay of the PXL and Exu exo-like (PXL: blue, Exu:Tan) and SAM-like (PXL: purple, Exu:pink) domains. The SAM-like domains differ in their orientation with respect to their Exo-like domains. **(B)** Overlay of the PXL SAM-like (purple) and Exu SAM-like (pink) domains showing conserved secondary structure alignment. **(C)** A short loop between the α8 and α9 helices causes the PXL SAM-like domain to orient itself differently than the Exu SAM-like domain.

**Supplementary figure S3: PXL selectively binds poly(UG) RNA sequences utilizing positively charged residues on both the SAM-like and Exo-like domains.**

**(A)** Fluorescence polarization (left) and electrophoretic mobility shift assays (right) of all DNA constructs used to assess PXL binding. **(B)** Fluorescence polarization (left) and electrophoretic mobility shift assays (right) of all RNA constructs used to assess PXL binding and the PXL 2K1R/3D binding deficient mutant.

**Supplementary figure S4: Representative images of U2OS cells used for RNA-seq and immunofluorescence. (A, B)** Representative image channels of individual cells utilized for RNA-sequencing and immunofluorescence. Both **(A)** YFP-PML-1 expressing cells and **(B)** YFP-PML-1(1-600) form nuclear bodies. **(C)** Comparison of the WT and ∆PXL protein expression levels based on nuclear YFP fluorescence. Corrected cell total fluorescence (CTCF) for YFP-PML-1 and YFP-PML-1(1-600), significance defined as p < 0.05.

**Supplementary figure S5: PXL impacts gene expression of known PML-NB interacting GO pathways. (A)** K-means clustering of all protein-coding genes detected in bulk-RNA sequencing. Expression was measured relative to the PML-KO strain. Log_2_FC were normalized to the mean of WT, PML-1, and ∆PXL. Clusters demonstrating gene expression changes in a PXL dependent manner are depicted in the inset (upregulated and downregulated). **(B, C)** GO analysis of genes specifically upregulated or downregulated by PXL.

**Supplementary figure S6: Transcriptome changes of all cell lines relative to PML-KO cells. (A)** Volcano plot of RNA abundance changes in cell expressing PML-1∆PXL vs PML-1 and **(B, C)** GSEA analysis. **(D)** Volcano plot of RNA abundance changes in cell expressing PML-1 vs PML-KO and **(E, F)** GSEA analysis. **(G)** Volcano plot of RNA abundance changes in cell expressing PML-1∆PXL vs PML-KO and **(H, I)** GSEA analysis. **(J)** Volcano plot of RNA abundance changes in cell expressing all PML isoforms (U2OS) vs PML-KO and **(K, L)** GSEA analysis.
